# The Sources of Strength Australia Project: study protocol for a cluster randomised controlled trial

**DOI:** 10.1186/s13063-016-1475-1

**Published:** 2016-07-26

**Authors:** Alison L. Calear, Jacqueline L. Brewer, Philip J. Batterham, Andrew Mackinnon, Peter A. Wyman, Mark LoMurray, Fiona Shand, Dominique Kazan, Helen Christensen

**Affiliations:** 1 Centre for Mental Health Research, Research School of Population Health, The Australian National University, 63 Eggleston Road, Acton ACT 2601, Canberra, Australia; 2 Orygen, The National Centre of Excellence in Youth Mental Health, University of Melbourne, Melbourne, Australia; 3 Department of Psychiatry, University of Rochester School of Medicine and Dentistry, Rochester, NY USA; 4 Sources of Strength, Bismarck, ND USA; 5 Black Dog Institute, University of New South Wales, Sydney, Australia

**Keywords:** Adolescent, Prevention, Help-Seeking, School, Suicide, Mental health

## Abstract

**Background:**

The school system has been identified as an ideal setting for the implementation of prevention and early intervention programs for suicide. However, in Australia, suicide-prevention programs that are routinely delivered in the schools are lacking. Internationally, evidence exists for the effectiveness of peer-led interventions that take a social connectedness approach to improve help-seeking for suicide. The aim of the current trial is to test the effectiveness of the Sources of Strength program to promote help-seeking for suicide in adolescents in Australian high schools.

**Methods/design:**

This study is a two-arm, cluster-randomised, controlled trial that will compare the evidence-based Sources of Strength program to a wait-list control condition. Sixteen Australian high schools will be recruited to the trial, with all adolescents in years 7 to 10 (12–16 years of age) invited to participate. Peer leaders from intervention-condition schools will receive training in the Sources of Strength program and will integrate positive messages, across 3 months, with the support of adult advisors. Activities may take the form of class presentations, posters, videos, and messages on social media sites and will aim to change help-seeking norms, strengthen youth-adult connections, and promote positive coping. The primary outcome measure for the study is help-seeking intentions, whereas secondary outcomes include help-seeking behaviour, help-seeking attitudes and norms, referral of distressed peers, availability of adult help, positive coping, and suicidal behaviour. Data will be collected pre-intervention, post-intervention (after the initial 3 months of messaging), and at the end of the first (6-month follow-up) and the second year after implementation (18-month follow-up). Primary analyses will compare changes in help-seeking intentions for the intervention condition relative to the wait-list control condition using mixed-effect repeated-measures analyses to account for clustering within schools.

**Discussion:**

If proven effective, this universal social connectedness program for suicide could be more widely delivered in Australian high schools, providing a valuable new resource. The Sources of Strength program has the potential to significantly contribute to the mental health of young people in Australia by improving help-seeking for suicide. The findings from this research will also contribute to the evidence-base for peer-leadership programs internationally.

**Trial registration:**

Australian New Zealand Clinical Trials Registry, ACTRN12616000048482. Registered on 19 January 2016.

**Electronic supplementary material:**

The online version of this article (doi:10.1186/s13063-016-1475-1) contains supplementary material, which is available to authorized users.

## Background

Youth suicide is a significant public health problem. In 2013, suicide was the leading cause of death in young Australians aged 15–24 years. For Australian males aged 15–19 years, the annual suicide rate was 14.3 per 100,000 people and accounted for 34.8 % of total deaths, whereas for Australian females, the suicide rate was 5.6 per 100,000 people and accounted for 26.1 % of total deaths [1]. This equates to more than 12 suicides per month across Australia in this age group. Suicidal ideation and attempts among young people are also a significant concern. In a systematic review of suicide phenomena in young people worldwide, the mean proportion of adolescents reporting a lifetime suicide attempt or suicidal thoughts was 9.7 % and 29.9 %, respectively [2].

The significant burden associated with suicide is evident from the fiscal and societal costs associated with youth suicidal behaviour (ideation, attempts, and suicide deaths) such as emotional and psychosocial morbidity, medical care, lost productivity, and the secondary distress caused to family members and friends [3]. In 2003, suicide accounted for 21 % of total years of life lost for Australians aged 15–24 years and 7.1 % of disability adjusted life years for males of the same age. Non-fatal suicidal behaviour was also related to substantial disability and loss of years of healthy life [4].

The prevalence of youth suicide, and the significant burden associated with it, has given rise to the development of a range of interventions aimed at the prevention of suicidal behaviour and the promotion of help-seeking and early identification for suicide. The need to promote and assist help-seeking behaviour among youth is critical, as young people often do not seek or receive help for suicidal thoughts and behaviour [5–8]. Interventions for youth suicide prevention have been implemented in schools, communities and healthcare systems and are designed to reduce risk factors for suicidal behaviour or to identify individuals at risk and provide pathways to treatment or support [5, 9]. Schools have been identified as an ideal setting for the implementation of these interventions, as they provide a cost-effective and convenient way of reaching young people who spend a significant period of their time there, and schools are a key social setting for identifying problems and offering support [7, 10]. Depending on the approach employed, these programs can be delivered universally, to all students or staff in a school or classroom setting, or to a selective (at-risk) or indicated (early signs) group of students or staff [9].

Reviews of school-based suicide prevention interventions [5, 9, 11, 12] conclude that the evidence-base for existing programs is limited, with the need for more rigorous evaluations, randomised controlled trials and the inclusion, where feasible, of suicidal behaviour outcomes. One of the promising suicide prevention programs identified in the reviews of school-based programs [9] is the universal Sources of Strength peer leadership program [10, 13], which takes a social connectedness approach to improving help-seeking for suicide and general psychological distress. Social isolation and thwarted belongingness have been identified as risk factors for suicide [14]. The Sources of Strength program is designed to build socioecological protective influences across an entire school student population and focuses on enhancing help-seeking norms, youth-adult communication, and coping skills to promote help-seeking [10, 13].

With the support of adult mentors, peer leaders from diverse social groups are trained to conduct whole-school messaging activities that are intended to change peer-group norms, attitudes and behaviours. The program harnesses the social networks of the peer leaders to disperse the program’s messages. More specifically, the peer leaders are taught to model and encourage friends to (a) reinforce and create an expectancy that friends ask adults for help for suicidal friends, thereby increasing the acceptability of seeking help and reducing implicit suicide acceptability; (b) name and engage ‘trusted adults’ to improve communication and connections between youth and adults; and (c) identify and use interpersonal (e.g. family and positive friends) and formal coping resources (e.g. mental health services and positive activities) to promote healthy coping attitudes. An integral part of the program is the identification and utilisation of eight key protective factors, referred to as ‘sources of strength’. These sources encompass family support, positive friends, caring adults, positive activities, generosity, spirituality, mental health access, and medical access. Overall, the program acts to reduce suicidal behaviours by more effectively connecting suicidal youth with capable adults and prevent the development of suicidal behaviour by promoting positive coping for psychological distress (e.g. depression and anxiety) [10, 13].

The Sources of Strength program has been evaluated in a randomised controlled trial of 18 high schools, 453 peer leaders, and 2,675 students located in the USA [10]. Peer leaders in intervention schools undertook whole-school messaging over a 3-month period, with pre- and post-messaging questionnaires conducted. Consistent evidence was found for a positive intervention effect on the norms, attitudes, and behaviour of both peer leaders and the wider student population. In particular, training improved the peer leaders’ adaptive norms regarding suicide (Cohen’s *d* effect size = 0.34–0.75), their connectedness to adults (*d* = 0.49–0.62), and their school engagement (*d* = 0.22). Peer leaders also reported increased support to peers (*d* = 0.34) and a greater connection of distressed peers to adults (*d* = 0.21), with peer leaders referring suicidal friends to an adult four times more than untrained peer leaders in control schools. Among the wider student population, the program increased perceptions of adult support for suicidal youth (*d* = 0.63) and the acceptability of seeking help (*d* = 0.58). The perception of adult support increased the most in students with a history of suicidal ideation. The fidelity of the intervention was also assessed through school-based staff interviews, finding that 97.2 % of staff had observed or received intervention-specific messages, and 88.9 % of those named as ‘trusted adults’ had been contacted by a peer leader as the intervention had intended. Overall, the changes observed in the intervention schools were congruent with the immediate goals of the Sources of Strength program, which were to enhance norms pertaining to suicide help-seeking, increase knowledge of capable adults, and increase acceptability of engaging adults for help within student peer groups.

The program promotes help-seeking for suicide by changing norms about help-seeking for suicide, which traditionally has seen young people not seeking help from adults due to negative beliefs about professional help, ‘codes of silence’ (shame and self-stigma, fears about disclosure, and a lack of trust that others will understand) and attitudes promoting self-reliance [6, 7]. Peer leaders recruited to the program work to change these norms by promoting and modelling help-seeking behaviour and positive communication with ‘trusted adults’, as well as the utilisation of multiple sources of strength in times of distress, discouraging self-reliance. These actions work to normalise help-seeking behaviour and increase its acceptability [3, 8].

The project represents the first evaluation of the Sources of Strength program within the Interpersonal Theory of Suicidal Behavior. This theory posits that three key interpersonal constructs are central to suicidal behaviour: thwarted belongingness, perceived burdensomeness, and acquired capability for suicide [15]. The combination of thwarted belongingness and perceived burdensomeness is strongly associated with the development of suicidal desire, whereas serious suicidal behaviour only occurs when acquired capability for suicide is also present [14–16]. The Sources of Strength program has been postulated to act to reduce thwarted belongingness through its promotion of youth-adult connections and that the utilisation of Sources of Strength will promote positive coping, effectively reducing social isolation and encouraging reciprocally caring relationships.

### Aims of the trial

#### Primary aim

The primary aim of the trial is to test the effectiveness of the Sources of Strength intervention for increasing help-seeking intentions for suicide at post-intervention.

#### Secondary aims

The secondary aims are as follows:

1. To test the effectiveness of the Sources of Strength intervention for increasing help-seeking intentions for suicide at 6-month follow-up
2. To test the effectiveness of the Sources of Strength program for (a) increasing actual help-seeking behaviour and the usefulness of adult help, (b) improving attitudes and norms towards adult help, (c) greater referral of suicidal peers, and (d) increased availability of adult help, at post-intervention and 6-month follow-up
3. To test the effectiveness of the Sources of Strength program for increasing positive coping (mental wellbeing, Sources of Strength (SoS) coping, social support, and mastery) at post-intervention and 6-month follow-up
4. To test the effectiveness of the Sources of Strength program for reducing suicidal thoughts and behaviours (plans and attempts) at post-intervention and 6-month follow-up
5. To test the impact of the Sources of Strength program on thwarted belongingness at post-intervention and 6-month follow-up
6. To test if the Sources of Strength intervention effects are increased at 18 months following the second year of program implementation

#### Subsidiary aims

Subsidiary aims include the following:

1. Levels of student and staff exposure to Sources of Strength whole-school messaging will also be explored, including a *social network* analysis in a subset of intervention schools to determine whether peer leader messaging increases the density of positive social ties across students and increases positive connections to adults, particularly among isolated students. The social network investigation will also test engagement with social media messages posted by peer leaders at the intervention schools, accounting for both virtual and self-reported social networks within each school.
2. Data collected from the study will also be used to test models of *suicide risk*, including the Interpersonal Theory of Suicidal Behavior [14, 15] and the relationship between positive future thinking and suicide risk [17]. These models will explore a range of potential risk and protective factors, such as perceived burdensomeness, thwarted belongingness, acquired capability for suicide, entrapment, self-harm, depressive and anxiety symptoms, personality, and risk-taking. Analyses will be conducted cross-sectionally and prospectively.
3. Lastly, *predictors and mediators* of intervention effects and intervention engagement will also be explored, including demographic characteristics, suicide stigma, suicide literacy, suicidal ideation, depressive symptoms, bullying, school inclusion, risk taking and personality profiles, peer leader status and exposure to messaging. These analyses will also investigate subgroup effects, such as whether there are greater effects on help seeking intentions and coping among students with suicidal ideation compared to those without.

#### Trial hypotheses

At post-intervention and 6-month follow-up, participants in those schools receiving the Sources of Strength program, relative to participants in the wait-list control schools, will have:

Increased help-seeking intentions for suicide
(a) Increased actual help-seeking behaviour for suicide, more positive attitudes and norms associated with adult help for suicide, increased referral of suicidal peers, and greater perceived availability of adult help for suicidal peers
(b) Increased positive coping, which is demonstrated by higher mental wellbeing, greater endorsement of SoS coping, more positive social support and higher mastery
(c) Lower levels of suicidal ideation, plans, and attempts
(d) Decreased levels of thwarted belongingness, thus reducing suicide susceptibility

Furthermore:

2. (e) Within-school effects at intervention schools will be stronger at 18 months (as the messaging program matures) than at 6 months.

## Methods/design

### Study design

This study will be implemented as a two-arm, cluster-randomised controlled trial (RCT), with an intervention condition (Sources of Strength program) and 24-month wait-list control condition. Measurements will be taken on four occasions: pre-intervention, post-intervention (after 3 months of messaging), and in two follow-ups at the end of each school year (approximately 6- and 18-month follow-up). This study was granted ethical approval by the Australian National University Human Research Ethics Committee (protocol number 2015/199) and the individual State and Territory education departments (Catholic Education Office Archdiocese of Canberra & Goulburn, ACT Government Education and Training Directorate, and the NSW Government Department of Education) responsible for schools in the recruitment districts.

### Recruitment

It is planned to recruit 16 schools from the Australian Capital Territory (ACT) and New South Wales (NSW), Australia, through letters of invitation and direct contact from the research team. All students aged 12–15 years in participating schools will be invited to participate in the research, while a select group of students representing each year level (7–9) from the intervention schools will also be invited to undertake the Sources of Strength peer-leadership program. Students will be invited to peer-leadership positions based on referrals from school staff identifying their influence among their peer groups. Information and consent forms will be distributed to all students and their parent/guardian prior to the trial commencing, with written or verbal informed consent required from both. All students in the intervention schools will potentially be exposed to the school-based messaging undertaken by the peer leaders. However, only consenting students will complete the trial questionnaires. All peer leaders will require consent to participate in the Sources of Strength peer leadership training and to complete the trial questionnaires. Adult advisors in the intervention schools will also receive training to assist and support the peer leaders in their role and will receive ongoing support through webinars hosted by the US Sources of Strength team.

### Randomisation

Each participating school (cluster) will be randomised to the intervention or wait-list control condition. Cluster randomisation will be undertaken for administrative convenience, to avoid contamination of the wait-list control condition and for the ecological validity of providing the intervention at the school level. School randomisation will be carried out by a statistician not involved in the day-to-day conduct of the trial according to ICH Guidelines [18]. As whole schools will be allocated to a single condition (cluster design), a minimisation approach [19, 20] will be used to ensure balance across conditions on the basis of school location (ACT/NSW), school type (private/public), number of students, and sex ratio.

### Procedure

Prior to the commencement of the project in each school, trial liaison managers located in the ACT and NSW will meet with each school to introduce the project to the wider school staff population and to prepare specific clinical protocols within each school. These protocols will detail the processes that will be undertaken if a student reports high levels of suicidality in the trial questionnaires. Student questionnaires will be viewed by the trial liaison managers following their completion to identify ‘at-risk’ respondents. School psychologists, counsellors, or a nominated staff member will be notified of ‘at-risk’ students, and they will follow up with them in accordance with usual school procedures. Students will also be provided with help-seeking contacts (e.g. school psychologist/counsellor or general practitioner) and information (e.g. telephone helplines or websites).

Following the development of clinical protocols, schools allocated to the intervention condition will nominate school staff to act as adult advisors for the Sources of Strength program. The selected staff will be provided with training in the Sources of Strength program, and will undertake the nomination process for peer leaders. At the commencement of the study, all consenting students in participating schools (both intervention and control) will be invited to complete a pre-intervention questionnaire. Questionnaire completion will be co-ordinated by the trial liaison managers to ensure standardisation and student privacy. Questionnaires will be delivered during school time and will be completed online or via paper and pencil, depending on school preference and resourcing. The assessments will take approximately 30 to 40 minutes to complete and have been designed to reduce participant burden. All data will be securely stored at The Australian National University, with access to the data restricted to trial personnel and investigators.

Following completion of the pre-intervention questionnaire, students in intervention condition schools who have been nominated as peer leaders will undertake the Sources of Strength peer-leadership training (4–6 hours over 1 day). Following this training, the peer leaders will meet with the adult advisors in their school on a fortnightly basis to plan and undertake 3 months of whole-school messaging. Students in the wait-list control condition schools will undertake usual school activities during this period.

At the completion of the messaging phase, the post-intervention questionnaire will be administered to all students. A short survey will also be administered to all staff in intervention condition schools to assess the reach of the whole-school messaging and youth-adult connections. A 6-month follow-up questionnaire will also be administered to all students towards the end of the first year of the program.

**Fig. 1:**
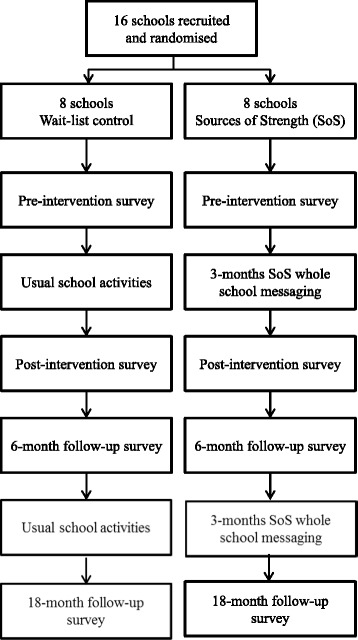
Flow of participants

In the second year of the project, a refresher Sources of Strength peer leadership training will be provided, with new adult advisors and peer leaders invited to participate alongside previous participants. This will be followed by a second 3-month whole-school messaging phase. A final 18-month follow-up questionnaire will be conducted at the end of the second year. After the completion of all questionnaires, schools in the wait-list control condition will be offered the Sources of Strength program, including the training of adult advisors and peer leaders, but questionnaires will not be administered. Figure 1 presents the flow of participants in the trial (See Additional file 1: Attachment A for SPIRIT Checklist).

### Intervention

The Sources of Strength program will consist of four phases in the first year of implementation. The first phase will be school and community preparation and will involve a 1-hour presentation to all school staff, the nomination and training (4–6 hours) of 2–3 staff members per school to act as adult advisors, and the development of a clinical protocol that outlines the school’s response to suicidal behaviours. The role of adult advisors will be to support and guide the peer leaders to conduct safe suicide prevention messaging.

The second phase of the program will be the nomination and training of peer leaders. Schools will be invited to nominate 2–10 % (maximum of 50 students) of their students as peer leaders, selecting key opinion leaders in diverse groups. The focus of peer leader training (4–5 hours) will be on interactive learning about the eight protective ‘sources of strength’, skills for increasing these resources for themselves and others in times of need, and engaging ‘trusted adults’ to help distressed and suicidal peers. The training program will focus on positivity, influence, and leadership, rather than purely on suicide prevention.

The third phase of the intervention will be whole-school messaging, which will entail activities to increase connectedness and will be undertaken over a 3-month period. In the first year, the messaging will involve (1) engaging ‘trusted adults’ by contacting them and acknowledging them as such; (2) encouraging friends to identify their ‘trusted adults’ and sharing these on a ‘wall of trust’; (3) undertaking pre-rehearsed classroom presentations to spread the sources of strength message by modelling how they have overcome adversity using positive coping and how they would engage a trusted adult for support for a suicidal friend; and (4) wider school ‘hope, help, and strength’ messaging using posters, public service announcements, videos and messages posted on social media sites (creating their own materials or using those available on the Sources of Strength Facebook page) to further disseminate the messages of the Sources of Strength program. Messaging will be empowering and strength-based, avoiding trauma, shock, or negative messaging, which could instil hopelessness. Peer leaders will meet fortnightly with the adult advisors to plan and approve messaging activities.

The final phase will be a celebration to recognise the peer leaders for their roles and accomplishments. In the second year, the program will be continued with the addition of new adult advisors and peer leaders alongside previous participants, and a repeat of the whole-school messaging. The reach and sophistication of messaging is expected to increase in the second year, as peer leaders become more comfortable and confident in their role. The wait-list control condition will continue usual school activities during the intervention phase of the trial and will receive the program after the follow-up periods.

### Assessments

Table 1 presents the scales that will be administered at each measurement occasion in the Australian Sources of Strength trial. It is noted that the measures included are relevant to different, sometimes multiple, study aims. All measures have previously been evaluated with adolescent samples and have good psychometric properties.

**Table 1:** Questionnaire scales for the Sources of Strength trial

	Pre-intervention	Post-intervention	6-month follow-up	18-month follow-up	No. of items
Demographics	✓	✓	✓	✓	6
Help-seeking Measures					
Adapted GHSQ	✓	✓	✓	✓	11
Adapted AHSQ	✓	✓	✓	✓	12
Help-seeking from adults	✓	✓	✓	✓	4
Help-seeking from adults at school	✓	✓	✓	✓	4
Reject codes of silence	✓	✓	✓	✓	4
Adult help for suicidal youth	✓	✓	✓	✓	4
Trusted adults at school	✓	✓	✓	✓	4
Referral of distressed peers	✓	✓	✓	✓	5
Positive coping					
SWEMWBS	✓	✓	✓	✓	7
SoS coping	✓	✓	✓	✓	8
Social support	✓	✓	✓	✓	15
Mastery	✓	✓	✓	✓	7
Suicidality					
YRBS	✓		✓	✓	4
SIDAS	✓	✓	✓	✓	5
Suicide risk models					
INQ	✓	✓	✓	✓	15
ACSS	✓	✓	✓	✓	7
Entrapment	✓	✓	✓	✓	4
Risk and protective factors					
Self-harm	✓	✓	✓	✓	3
SOSS	✓	✓	✓	✓	16
LOSS	✓				12
DQ5	✓	✓	✓	✓	5
MDI	✓	✓	✓	✓	13
SCARED-GAD	✓	✓	✓	✓	9
Bullying	✓	✓	✓	✓	3
Peer integration at school	✓	✓	✓	✓	4
School identification	✓				2
SURPS-23	✓				23
RTSHIA (RT)	✓	✓	✓	✓	8
Social network variables					
No. of friends	✓	✓	✓	✓	3
No. of trusted adults	✓	✓	✓	✓	2
Naming trusted adults	✓	✓	✓	✓	9
Naming close friends	✓	✓	✓	✓	21
Intervention engagement					
Intervention reach^a^		✓		✓	11
Fidelity questions^b^		✓		✓	6

*ACCS* Acquired Capability for Suicide Scale, *AHSQ* Actual Help-Seeking Questionnaire, *DQ5* Distress Questionnaire-5, *GHSQ* General Help-Seeking Questionnaire, *INQ* Interpersonal Needs Questionnaire, *LOSS* Literacy of Suicide Scale, *MDI* Major Depression Inventory, *RTSHIA* (*RT*) Risk Taking and Self-Harm Inventory for Adolescents – Risk-Taking Scale, *SCARED*-*GAD* Screen for Child Anxiety Related Emotional Disorders, *SIDAS* Suicidal Ideation Attributes Scale, *SOSS* Stigma of Suicide Scale, *SoS* Sources of Strength, *SURPS*-*23* Substance Use Risk Profile Scale, *SWEMWBS* The Short Warwick-Edinburgh Mental Well-being Scale, *YRBS* Youth Risk Behaviour Survey

^a^Only students in intervention schools respond to these questions

^b^Only peer leaders in intervention schools respond to these questions

### Demographic variables

The following demographic variables will be measured: age, sex, grade, language spoken at home, and school name.

### Help-seeking

The primary aim of the Sources of Strength program is to promote help-seeking from a trusted adult in times of distress. A series of measures are therefore included in the questionnaires that assess help-seeking intentions, help-seeking behaviour, and the perceived usefulness of adult help, attitudes towards adult help, the referral of distressed peers, and the availability of adult help (for suicidal youth and more generally). The adapted version of the *General Help*-*Seeking Questionnaire* (GHSQ) [21, 22] is the primary outcome measure in the current study. It assesses intentions to seek help for personal or emotional problems from 11 different formal and informal sources (e.g. friend, parent, psychologist, or teacher). Respondents indicate how likely they are to seek help from each of the sources on a scale ranging from 1 (extremely unlikely) to 7 (extremely likely).

The adapted *Actual Help*-*Seeking Questionnaire* (AHSQ) [22, 23], and subsequent help-seeking measures, are secondary outcome measures that further explore the impact of the program on help-seeking. The AHSQ assesses recent actual help-seeking behaviour and consists of the same 11 sources of help as the GHSQ, in which the respondent either does or does not report having sought help from for a mental health problem in the past 3 months. In addition to the AHSQ, *actual help*-*seeking from adults* is measured by four items (yes/no) that assess the experience of help-seeking from adults, conditional on responses to the AHSQ. If students indicate that they have sought help from at least one adult for a mental-health problem on the AHSQ, then four questions will be presented asking whether any of the adults that they have had contact with have made them feel supported, helped them get through the situation, made the situation worse, or made them more likely to seek further help from adults. These items were drawn from the previous US Sources of Strength trial [10].

Help-seeking attitudes are assessed by two measures including the *help*-*seeking from adults at school* [24], which is a four-item measure derived from the previous US Sources of Strength trial. The measure assesses attitudes and perceived norms about seeking help from adults at school on a scale from 1 (strongly disagree) to 4 (strongly agree). The items on this scale enquire as to whether, if really upset and needing help, a student would talk to a counsellor or other adult at school, whether they believe these adults could help, and whether friends and family would want them to seek help. Items on this scale are summed, with higher scores reflecting more positive help-seeking from adults at school. A second four-item scale (r*eject codes of silence*) will measure student attitudes toward overcoming secrecy barriers to engaging adult help for suicidal peers. Items are responded to on a four-point scale ranging from 1 (strongly disagree) to 4 (strongly agree), with higher scores indicative of more positive intentions to get help for suicidal friends and resist requests for secrecy [8, 10].

A further two scales, also from the previous US Sources of Strength trial [10], assess the availability of adult help. The first four-item scale specifically assesses the availability of *adult help for suicidal youth*, enquiring about whether youth know adults who could help a friend thinking of suicide, whether their school has people who can help students going through hard times, whether they can think of an adult they trust enough to help a suicidal friend, and whether students with problems can get help from adults in their school. Items on this scale are responded to on a four-point scale ranging from 1 (strongly disagree) to 4 (strongly agree). Higher scores are indicative of a perception of strong adult support being available for suicidal youth in the school. The second four-item scale assesses *trusted adults at school* [25]: i.e. adolescents’ perception of having an adult to turn to for open, honest, and safe communication. Items within this scale (e.g. ‘At my school there is an adult who listens to what I have to say’) are also rated on a four-point scale ranging from 1 (strongly disagree) to 4 (strongly agree), with higher scores suggestive of students having adults at school that they feel they can trust and talk to about problems.

Finally, five items will be administered to assess the respondent’s friends’ suicide risk and the *referral of distressed peers*. These questions assess help-seeking behaviour by friends considering suicide (e.g. ‘Have any of your friends told you they were thinking of killing themselves’) and referral of friends to help sources (e.g. ‘I told a friend to get help because of emotional or behaviour problems’). These items are adapted from the *Referred Distressed Peers to Adults Scale* [10] and from questions being used in the current US evaluation of the Sources of Strength program.

### Positive coping

A range of positive coping measures will also be collected as secondary outcomes. Mental wellbeing will be evaluated using the short *Warwick*-*Edinburgh Mental Well*-*being Scale* (SWEMWBS) [26, 27]. The SWEMWBS is a self-report scale that consists of seven positively worded items that measure different aspects of positive mental health. Each item is rated on a five-point Likert type scale ranging from 1 (none of the time) to 5 (all of the time), with a total scale score (7–35) calculated by transforming and then summing item scores, with higher scores indicating a greater level of mental wellbeing.

A further eight-item scale, drawn from the previous US Sources of Strength evaluation [10], will be included to specifically assess *Sources of Strength Coping*. These items measure the extent to which students view the eight resources targeted by the Sources of Strength program as useful to them in overcoming challenges in their life. These resources cover both formal (e.g. access to mental health) and informal resources (e.g. family). Items on this scale are responded to on a four-point scale ranging from 1 (strongly disagree) to 4 (strongly agree), with higher scores indicating greater coping.

Social support will additionally be measured using the supportive interactions and negative interactions questions from *Schuster*’*s Social Support Scale* [28]. Fifteen items will be used to measure both positive and negative interactions with friends, family members, and teachers (in place of the original ‘spouse’ category), rated on four-point Likert scale ranging from ‘often’ to ‘never’. Scores are interpreted per category, for friends, family members, and teachers. Higher scores on the supportive interactions scales are indicative of more supportive interactions, and higher scores on the negative interactions scales indicate more negative interactions.

Coping mastery, or the belief that life is under an individual’s control rather than fatalistically determined, will also be assessed using the seven-item *Pearlin Mastery Scale* [29]. Items on this scale are rated on a four-point scale ranging from 1 (strongly disagree) to 4 (strongly agree). Negatively worded items require reverse coding prior to scoring, with higher scores indicating greater levels of mastery.

### Suicidality

Suicidality is a key secondary outcome, as one of the long-term goals of the Sources of Strength program is to prevent and reduce suicidal thoughts and behaviours, after the cessation of the program, through the promotion of help-seeking and positive coping. The *Youth Risk Behaviour Survey* (YRBS) [30] is a four-item (yes/no) measure that will be administered to assess if the respondent has had suicidal ideation or made a suicide plan or attempt (and its seriousness) during the past 12 months. Research indicates that the measurement of suicidal behaviours in adolescents is acceptable and does not pose an iatrogenic risk [31, 32]. Suicidal ideation will be further investigated in the current study using the five-item *Suicidal Ideation Attributes Scale* (SIDAS) [33]. Items on this scale are rated on ten-point scale and assess the frequency of, and control over, suicidal thoughts. Higher scores on the SIDAS are indicative of greater suicidal ideation.

### Suicide risk models

A subsidiary aim of the current study is to explore suicide risk models in an adolescent population to better understand the development and progression of suicidality in young people. The *Interpersonal Needs Questionnaire* (INQ) [34] and the *Acquired Capability for Suicide Scale* (ACSS) [35] will be administered to assess the three constructs (thwarted belongingness, perceived burdensomeness, and acquired capability for suicide) that comprise the Interpersonal Theory of Suicidal Behavior. The INQ contains 15 statements that assess thwarted belongingness (nine items) and perceived burdensomeness (six items). Thwarted belongingness refers to social isolation and broadly comprises loneliness and the absence of reciprocally-caring relationships, while perceived burdensomeness consists of two key dimensions of interpersonal functioning: cognitions of self-hatred and beliefs that one is a liability to others (20). Each item of the INQ is responded to on a seven-point scale ranging from 1 (not at all true for me) to 7 (very true for me). A total scale score for each sub-scale can be calculated by summing individual items for each scale (after reverse coding six items), and a total scale score is calculated by summing these subscale scores. Total scale scores can range from 9 to 63 for thwarted belongingness and 6 to 42 for perceived burdensomeness, with higher scores on each sub-scale indicating higher levels of thwarted belongingness and perceived burdensomeness.

The *Acquired Capability for Suicide Scale* (ACSS) [35] contains seven items that assess fearlessness about death. Each item is responded to on a four-point Likert-type scale ranging from 0 (not at all like me) to 4 (very much like me). Total scale scores are calculated by summing item scores and can range from 0 to 28. Higher scores are representative of greater levels of fearlessness about death.

The relationship between positive future thinking and suicide risk will also be explored through the assessment of *entrapment* [36]. Entrapment will be measured by a four-item scale [17], which was adapted from Gilbert and Allan [36]. Items on this scale are responded to on a five-point scale ranging from ‘not at all like me’ to ‘extremely like me’. Higher scores are suggestive of higher entrapment or a strong motive to take flight that is blocked (e.g. ‘I feel trapped inside myself’).

### Risk and protective factors

A range of putative risk and protective factors will also be measured to be explored as mediators or moderators of intervention effects and for inclusion in models of suicide risk. The factors explored will include self-harm, suicide stigma, suicide literacy, general psychological distress, depressive and anxiety symptoms, bullying, school social inclusion, personality, and risk taking behaviour.

*Self*-*harm* will be measured using three items (yes/no) that assesses the presence of self-injury, whether the intention of the self-injury was to experience pain or suffering and whether the intention of the self-injury was to die.

Suicide stigma will be investigated using the *Stigma of Suicide Scale* (SOSS) [37]. The SOSS contains 16 one or two-word descriptors of people who die by suicide and respondents are asked to rate their level of agreement with each item on a five-point scale ranging from ‘strongly disagree’ to ‘strongly agree’. Higher scores on this scale are suggestive of greater suicide stigma. The *Literacy of Stigma Scale* (LOSS) will also be administered [38] to assess respondent knowledge of the signs and symptoms of suicide, risk factors, and prevention and treatment options. The LOSS consists of 12 items on a ‘true/false/don’t know’ scale. Higher scores are reflective of greater suicide literacy.

General psychological distress will be assessed with the newly developed *Distress Questionnaire Scale* - *5* (DQ5) [39]. This scale contains five items rated on a five-point scale from 1 (never) to 5 (always), with total scores ranging from 5–25. The screener has been shown to be accurate in detecting a range of common mental health problems in community-based settings.

Depressive symptoms will be measured by the *Major Depression Inventory* (MDI) [40]. This scale consists of 12 items (10 questions with 2 sub-items) that cover the ICD-10 and DSM-IV symptoms of depression. These items are rated on a six-point scale from 0 (all the time) to 5 (at no time) with total scale score ranging from of 0 to 50. Higher scores indicate the presence of more depressive symptoms.

Generalised anxiety will be measured using the *Screen for Child Anxiety Related Emotional Disorders GAD subscale* (SCARED-GAD) [41]. The SCARED-GAD consists of nine items rated on a three-point scale ranging from 0 (not true or hardly ever true) to 2 (true or often true). The items included on the questionnaire are reflective of the DSM-IV criteria for generalised anxiety disorder in childhood.

Three items will be used to assess *bullying behaviour* online and in person. The items, which are being used in the current Sources of Strength trial in the USA, are based on questions by Klomek and colleagues [42]. The items enquire how often students have been bullied and how often they have bullied others, on a five-point scale ranging from 1 (not at all) to 5 (most days).

Four items, drawn from the current US Sources of Strength trial, will be used to assess *peer integration at school*, addressing both inclusion and isolation at school (e.g. ‘At my school I feel close to other students’). These items will be rated on a four-point scale ranging from 1 (strongly disagree) to 4 (strongly agree), with higher scores indicating a higher level of social support at school. An additional two items will assess school identification (e.g. ‘I feel a strong connection with my school’) and will be rated on a seven-point scale ranging from 1 (not at all) to 7 (very much).

The *Substance Use Risk Profile Scale* (SURPS-23) [43] will be used to assess the four personality dimensions of hopelessness, anxiety sensitivity, impulsivity and sensation seeking. The SURPS-23 contains 23-items rated on a four-point scale ranging from ‘strongly disagree’ to ‘strongly agree’. The overall score sums to suggest personality risk for substance misuse.

Lastly, risk-taking will be measured using the *Risk*-*Taking and Self*-*Harm Inventory for Adolescents* [44]. This subscale contains eight-items regarding general risk taking behaviours for adolescents, such as classroom cheating, physical fights, excess alcohol consumption, and drug use, which are rated on a four-point scale ranging from ‘never’ to ‘many times’. Higher scores indicate greater participation in risky activities.

### Social network variables

A number of measures, developed specifically for this trial, will also be included to measure social networks and Sources of Strength intervention effects through these networks. These include the following:

1. *Number of friends* – Assessing the number of close friends at school and online (three items)
2. *Number of trusted adults* – Assessing the number of trusted adults at school and outside of school (two items)
3. *Naming trusted adults* – Listing the names of seven adults whom students would ask for help for a suicidal friend
4. *Naming close friends* – A sub-set of students will be invited to list up to seven of their closest friends, how likely they are to seek help from these friends, and how much they are influenced by them, which will be included in a social network analysis.

### Intervention engagement

*Intervention reach* will be measured by 11 questions (e.g. ‘Have you seen a presentation or assembly about strengths that help teens get through hard times?’). The peer leader groups in the intervention schools will additionally be asked six questions assessing the *fidelity* of the intervention. Example items include whether students held regular peer leader group meetings, and whether the peer groups organised any sources of strength activities. School staff in intervention schools will also be invited to participate in a short survey to assess the reach of the whole school messaging and youth-adult connections.

### Sample size and power calculations

Calculation of required sample size was based on detecting a post-intervention effect size of 0.33. This reflects the universal nature of this intervention and is based on effect sizes obtained in the US evaluation of the program [10]. Power was set at 0.8, α = .05 (two-tailed), and correlation of .5 assumed between baseline and endpoint scores. To allow for possible clustering effects (participants from the same school having characteristics and outcomes more alike than between schools), a design effect [45] was calculated to be 12.96, assuming an intraclass correlation (ICC) of 0.04 and an average school size of 300 students. The estimate of the ICC reflects previous Australian school-based studies that have found non-significant intraclass correlations (ICC = .02) [46] and from the US trial ICC = .04 [10]. Accommodating a 20 % attrition rate [46], the target sample size is set at 4,800 or 2,400 students across eight schools per condition.

### Statistical analysis

Analyses of continuous measures will be undertaken on an intent-to-treat basis, including all participants randomised regardless of treatment actually received or withdrawal from the study. Mixed-model repeated-measures analyses will be used because of the ability of this approach to include participants with missing data without using biased techniques, such as last observation carried forward [47]. In addition, by incorporating appropriate random effects for schools, this approach can accommodate and assess the magnitude and significance of clustering effects. The primary aim will be evaluated by a contrast examining change in help seeking intentions from pre-intervention to post-intervention in the Sources of Strength condition compared to that in the wait-list control condition. For suicide attempts and other dichotomous outcomes, a comparable binary mixed modelling approach [48] will be used. Differences in relative risk for incidence of suicide attempt from baseline to the 6- and 18-month follow-ups will be assessed. Mediators of intervention outcome including suicidal ideation, depressive symptoms and exposure to school-based messaging will be explored using interaction terms in mixed effects models and using latent class analyses [49]. Models of suicide risk will be developed using regression analyses and structural equation models, testing the Interpersonal Theory of Suicidal Behavior framework and examining the role of additional psychosocial and demographic risk factors. Social network factors will be assessed in a subset of two schools (one control and one intervention), evaluating up to seven connections per student at pre- and post-intervention, to estimate a range of network indices of density, reciprocity, clustering and individual centrality. Change in social network characteristics as a function of intervention condition will be tested. During the messaging phase, engagement with social media messages posted by peer leaders at the intervention schools will be analysed by categorising posts and assessing the types of messaging that best engage students by measuring reach through likes, shares, and comments. This sub-study measures ‘digital footprint’ behaviour, and thus complements the self-report measures of connectivity.

Given the broad scope of research questions and the incremental availability of data, the publication plan for the study is to examine the help-seeking outcomes, coping/suicidality outcomes, theoretical models, and social network analyses in separate publications. The primary report will be of post-intervention and 6-month follow-up outcomes, with subsidiary analyses to be conducted at the 18-month follow-up to investigate long-term outcomes for the second year of the program.

## Discussion

One of the key recommendations of the Australian Senate Community Affairs report on suicide in Australia was for further research into suicide prevention, including detailed evaluations of suicide prevention interventions [50]. The current project represents an opportunity to evaluate such a program, and contribute to the presently sparse evidence base for suicide prevention programs in Australian schools [11]. The need for such research is high, given the prevalence of suicidal behaviours in Australian youth and the significant burden associated with them [1, 4], as well as pressure on governments from the community to prevent youth suicide.

The current project aims to increase help-seeking for suicide in adolescents, which is an important outcome given the low rates of help-seeking behaviour for suicidal thoughts and behaviours currently exhibited in young people [5–8]. Increasing help-seeking behaviours is the cornerstone to preventing and reducing suicidal ideation, attempts and deaths. Given the successful dissemination of the program in multiple states across the US, the trial, if positive, will provide support for a practical intervention that has been demonstrated to translate to the population level. Suicide prevention in schools is underdeveloped in Australia and the program tested in this trial has the potential to significantly impact the suicide rate of young Australians and stimulate more high quality research in this critical area.

This is the first rigorous evaluation of a social connectedness intervention for suicide prevention in Australian schools, with a focus on increasing help-seeking behaviour for suicide through peer leader endorsement of positive help-seeking norms, youth-adult communication and the promotion of positive coping. The trial will also provide a novel opportunity to test the Interpersonal Theory of Suicidal Behavior within an adolescent population over two years, as well as evaluating the Sources of Strength program within this model for the first time. The Interpersonal Theory of Suicidal Behavior is a predominant theory of suicidal behaviours that aligns closely with the social connectedness approach of the program. The current trial will further add to the knowledge base of the Sources of Strength program, and suicide help-seeking research more generally.

### Trial status

Participants are currently being recruited to the trial.

## Abbreviations

ACCS, Acquired Capability for Suicide Scale; ACT, Australian Capital Territory; AHSQ, Actual Help-Seeking Questionnaire; *d*, Cohen’s d effect size; DQ5, Distress Questionnaire-5; GHSQ, General Help-Seeking Questionnaire; ICC, intraclass correlation; INQ, Interpersonal Needs Questionnaire; LOSS, Literacy of Suicide Scale; MDI, Major Depression Inventory; NSW, New South Wales; RTSHIA (RT), Risk Taking and Self-harm Inventory for Adolescents–Risk-taking Scale; SCARED-GAD, Screen for Child Anxiety Related Emotional Disorders–Generalised Anxiety Disorder; SIDAS, Suicidal Ideation Attributes Scale; SoS, Sources of Strength; SOSS, Stigma of Suicide Scale; SURPS-23, Substance Use Risk Profile Scale; SWEMWBS, The Short Warwick-Edinburgh Mental Well-being Scale; US, United States; YRBS, Youth Risk Behaviour Survey

## Additional file

Additional file 1: SPIRIT 2013 Checklist: Recommended items to address in a clinical trial protocol and related documents*. (DOC 121 kb)
